# On-Call Duty Effects on Sleep-State Physiological Stability in Male Medical Interns

**DOI:** 10.1371/journal.pone.0065072

**Published:** 2013-06-04

**Authors:** Yu-Hsuan Lin, Yen-Cheng Ho, Sheng-Hsuan Lin, Yao-Hsien Yeh, Chia-Yih Liu, Terry B. J. Kuo, Cheryl C. H. Yang, Albert C. Yang

**Affiliations:** 1 Department of Psychiatry, National Taiwan University Hospital, Taipei, Taiwan; 2 School of Medicine, Chang Gung University, Taoyuan County, Taiwan; 3 Department of Psychiatry, Chang Gung Memorial Hospital at Linkou, Taoyuan County, Taiwan; 4 Department of Epidemiology, Harvard School of Public Health, Boston, Massachusetts, United States of America; 5 Department of Internal Medicine, Ren-Ai branch, Taipei City Hospital, Taipei, Taiwan; 6 Institute of Brain Science, National Yang-Ming University, Taipei, Taiwan; 7 Sleep Research Center, National Yang-Ming University, Taipei, Taiwan; 8 Division of Psychiatry, School of Medicine, National Yang-Ming University, Taipei, Taiwan; 9 Department of Psychiatry, Taipei Veterans General Hospital, Taipei, Taiwan; Universidade Federal do Rio de Janeiro, Brazil

## Abstract

**Background:**

On-call duty among medical interns is characterized by sleep deprivation and stressful working conditions, both of which alter cardiac autonomic modulation. We hypothesized that sleep stability decreased in medical interns during on-call duty. We used cardiopulmonary-coupling (CPC) analysis to test our hypothesis.

**Methods:**

We used electrocardiogram (ECG)-based CPC analysis to quantify physiological parameters of sleep stability in 13 medical interns during on-call and on-call duty-free periods. There were ten 33.5-h on-call duty shifts per month for interns, each followed by 2 on-call duty-free days, over 3 months. Measurements during sleep were collected before, during, and after an on-call shift. Measurements were repeated 3 months later during an on-call duty-free period.

**Results:**

The medical interns had significantly reduced stable sleep, and displayed increased latency to the first epoch of stable sleep during the on-call night shift, compared to the pre-call and on-call duty-free nights. Interns also had significantly increased rapid-eye-movement (REM) sleep during the on-call night shift, compared to the pre-call and on-call duty-free nights.

**Conclusion:**

Medical interns suffer disrupted sleep stability and continuity during on-call night shifts. The ECG-based CPC analysis provides a straightforward means to quantify sleep quality and stability in medical staff performing shift work under stressful conditions.

## Introduction

Medical interns typically work the greatest number of hours per week among all types of hospital trainees [1]. On-call duty among medical interns is characterized by sleep deprivation and stressful working conditions. Sleep deprivation alters cardiovascular reactivity to acute stressors [2], and increases the incidence of cardiovascular disease (CVD) [3], [4], [5]. Systematic reviews have reported that such long working shifts and erratic schedules lead to acute and chronic sleep deprivation and poor sleep quality in training physicians, resulting in numerous adverse consequences in patient care [6]. Moreover, early in the academic year in a traditional extended-duty shift model, each new admission during the on-call shift is associated with a reduction in the amount of on-call sleep, and an increase in the total shift duration [7]. Recent prospective studies and meta-analyses also suggest that long working hours [8] and shift work [9] increase the risk of CVD. However, few studies have compared cardiac autonomic modulation among physicians in on-call work settings [10], [11], [12] or during on-call sleep periods [12].

Cardiopulmonary-coupling (CPC) analysis has recently been developed to quantify sleep quality and stability using an electrocardiogram (ECG)-based technique that measures heart rate variability (HRV) and an ECG-derived respiratory signal [13]. The CPC analysis generates a sleep spectrogram that demonstrates coupled sleep oscillations with spontaneously transitioning periods of high-frequency coupling that represent stable sleep, low-frequency coupling representing unstable sleep, and very-low-frequency coupling representing rapid-eye-movement (REM) sleep or awakening states. The CPC method has been used in the analysis of sleep apnea [13], [14], major depression [15], fibromyalgia [16], and heart failure [17] based solely on the continuous ECG signal [13], [14].

Because on-call duty is associated with reduced sleep stability, we hypothesized that such decreased sleep stability in medical interns may be quantified using the CPC analysis method. The aim of our study was to assess sleep stability in medical interns using ECG-based CPC analysis during on-call duty and on-call free-duty periods.

## Methods

### Participants

We recruited 13 medical interns at Chang Gung Memorial Hospital as study volunteers, each with 1 year of previous clinical training. We gave a 20-min PowerPoint presentation to all volunteers to outline the aims of our study. They were informed of our intention to obtain objective and subjective evaluations of their performance, assessments of their autonomic functioning, and measurements of their sleepiness and emotional state. All participants provided written informed consent.

The study ran from October 2007 to February 2008. The study protocol was approved by the Ethics Committee of Chang-Gung Memorial Hospital. All participants were men aged 25.3±1.9 years from the seventh grade of a medical-college student population. They did not abuse hypnotic drugs or alcohol, and did not use caffeine or nicotine during the entire period of study. None of them had any medical conditions known to involve sleep or the autonomic nervous system, such as psychiatric or cardiovascular diseases.

There were ten 33.5-h on-call duty shifts per month for interns, each followed by 2 on-call duty-free days, over 3 months. The on-call shift consisted of routine work from 7∶30 am to 5∶00 pm, followed by a 15-h on-call shift. For the sleeping periods, we divided the on-call cycle into the pre-call night, the on-call night, and the post-call nights. The assessments were conducted during the third month of the internal medicine course. After 3 months, the interns were evaluated for a second time during the third month of an on-call duty-free period, which provided control data for comparisons. The courses during the on-call duty-free period included nuclear medicine, pathology, or radiology. The on-call duty-free period was less demanding for interns because the residents served as the hospital’s first-line medical staff, whereas the interns served as the first-line medical staff during the on-call duty period.

### Continuous ECG Monitoring

**Figure 1:**
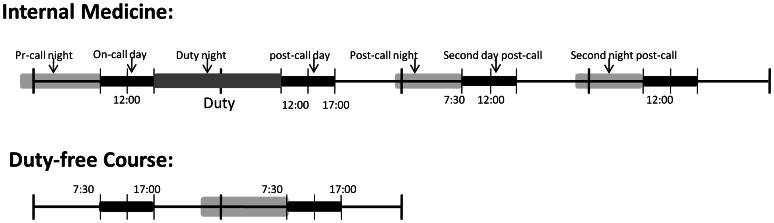
Study design. Participants were aware of this study’s intention to obtain evaluations of their autonomic functioning (cardiopulmonary coupling analysis, CPC). The on-call day comprised routine work from 7∶30 to 17∶00, followed by on-call duty for 15 hours. The on-call phase lasted for 4 days, one cycle after another; these were: the pre-call day, the on-call day, post-call day, and second day post-call. Measurements were obtained during the third month of the internal medicine course. After 3 months, the tests were repeated during the third month of a duty-free course and this was used as a self-control group.

Our study protocol is outlined in Fig. 1. The ECG data were obtained for all participants using a TD1 Miniature Physiological Signal Recorder (Taiwan Telemedicine Device, Taiwan) as previously described [18]. The small size (5.2×3.1×1.2 cm) and low weight (11 g) of the recorder produced minimal interference during both working and sleeping, resulting in minimal additional stress for the participants. Each intern was monitored by a two-lead ECG recorder with an accelerometer that was attached at 9∶00 pm prior to sleeping on the pre-call night. The device continuously recorded for 44 h until 5∶00 pm on the post-call day. The ECG and accelerometer monitoring was resumed for an additional 44 h from 9∶00 pm on the post-call night to 5∶00 pm on the third post-call day. After 3 months, the ECG monitoring was repeated during one on-call duty-free day.

### Sleep Data and Accelerometry

We obtained sleep data using a weekly sleep log and an accelerometer that was attached to the recorder [12]. Acceleration values were stored in the flash memory for each axis, as X (mediolateral), Y (vertical), and Z (anteroposterior). Each axis had a sampling frequency of 125 Hz, and could detect accelerations ranging from −3 to 3 *g*. A vectorial magnitude was calculated as √X^2^+Y^2^+Z^2^. The quantified magnitude of physical activity was estimated by calculating the root mean square of the vectorial magnitude for each period (epoch). We applied the previously described zero-crossing method to count the number of times per epoch of sleep time that the activity signal level crossed near zero, 0.004087 *g* [16]. In our study, only the ECGs recorded during nocturnal sleep were analyzed.

### Cardiopulmonary Coupling Analysis

The autonomic nervous system has predictable characteristics that vary according to sleep depth and type [19], [20]. The CPC analysis is derived from an estimation of the coupling between the autonomic and respiratory drives, using heart rate and respiratory modulation of QRS amplitude, respectively. This dual information can be extracted from a single channel of the ECG [13]. The ECG-derived respiratory signal has been described in detail [21], and is highly correlated with the actual respiration waveforms [22]. The algorithm of CPC analysis involves the extraction of the heart rate and the respiration waveforms from the ECG signal and the subsequent determination of sleep state based on the estimation of the cross-spectral power and coherence between the ECG-derived respiration and the heart rate signals. The data are analyzed in 128-s increments across a 512-s analysis window, progressing until the entire time series is analyzed.

The 3 physiological sleep states that are derived from CPC analysis are (1) stable, (2) unstable, and (3) REM sleep or the awakening state [13]. Physiologically stable sleep is associated with high-frequency coupling between heart rate and respiration at frequencies of 0.1 to 0.4 Hz. In contrast, physiologically unstable sleep is associated with low-frequency coupling between heart rate and respiration over a range of 0.01 to 0.1 Hz. The awakening state and REM sleep are associated with the presence of very-low-frequency coupling between heart rate and respiration below 0.01 Hz. Based on accelerometry data, REM sleep can be distinguished from the awakening state.

### Statistical Analysis

The heart rate was monitored repeatedly for each participant, and the value of heart rate measurements among different periods for the same person was highly correlated. Therefore, a generalized estimation equation was used to analyze the repeated measurements over time. All of the major determinants of the 3 sleep states were included in the statistical model, and the sleep data during the on-call duty-free period and pre-call night were treated as baseline data. The time points of the pre-call night, the on-call night, the post-call night, and the second-day post-call night represented categorical variables, demonstrating the effect of on-call duty, compared with the baseline sleep state. The variance-covariance pattern model was estimated to be heterogeneous-autoregressive in our study. The PROC MIXED procedure in the Statistical Analysis Software program, version 9.3 (SAS Institute, Cary, NC, USA), was used to fit the data. A *P* value of less than.05 was considered to represent a statistically significant difference.

## Results

**Figure 2:**
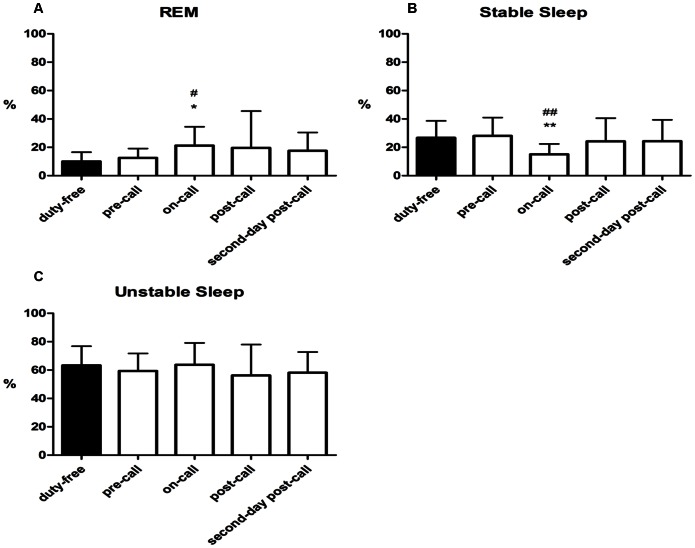
Patterns of sleep. (A) REM percentage of sleep on different days; (B) Stable sleep percentage of sleep on different days; (C) Unstable sleep percentage of sleep on different days; The results are expressed as Mean ± SD.^ ##^P<0.01 compared to duty-free day; ^#^P<0.05 compared to duty-free day; *P<0.05 compared to pre-call day; **P<0.01 compared to pre-call day by generalized estimation equation, n = 13.

**Figure 3:**
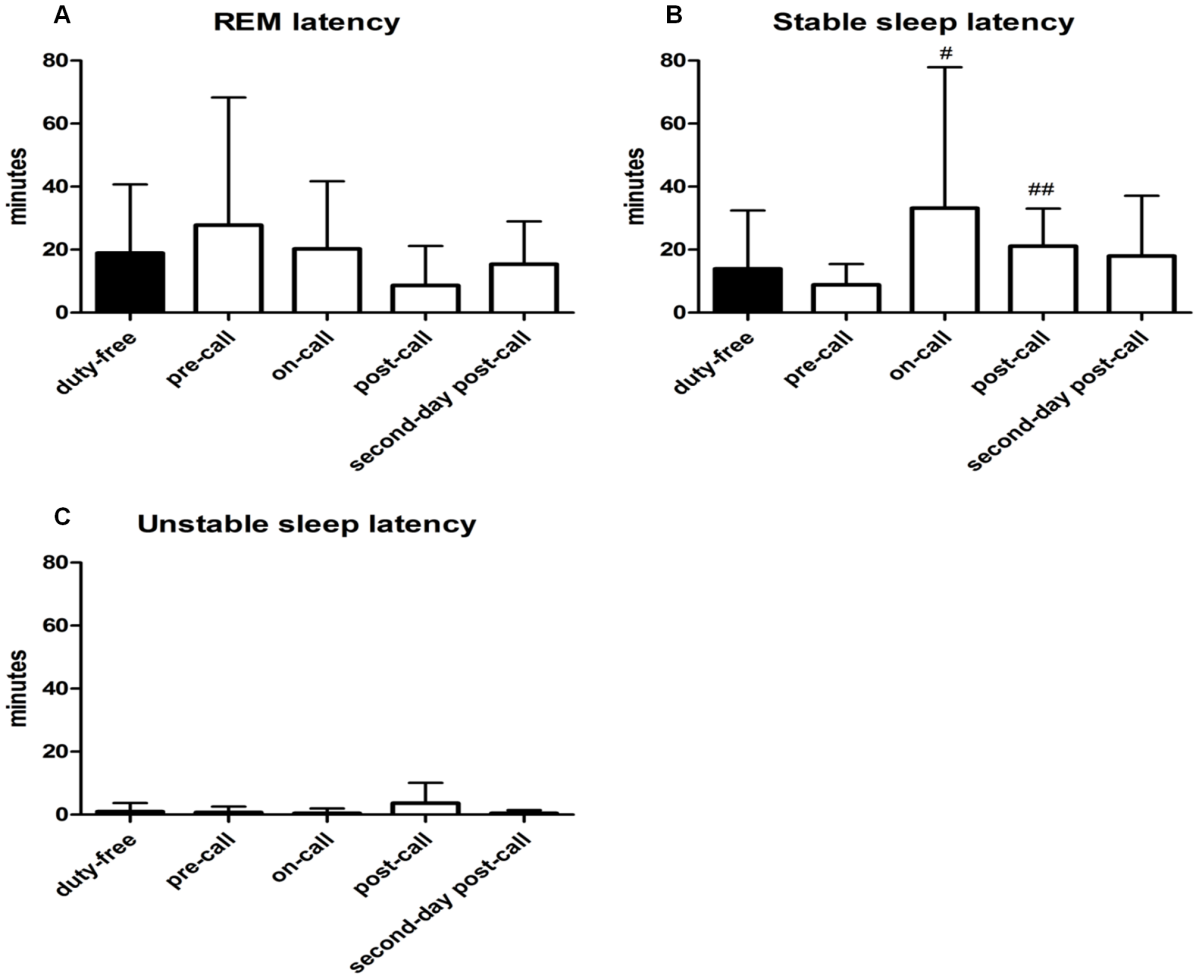
Latency to the first epoch. (A) REM latency on different days; (B) Stable sleep latency on different days; (C) Unstable sleep latency on different days; The results are expressed as Mean ± SD. ^##^P<0.01 compared to duty-free day; ^#^P<0.05 compared to pre-call day, by generalized estimation equation, n = 13.

The objective sleep indices derived from the CPC analysis are presented in Figs. 2 and 3. The periods of stable sleep significantly decreased during the on-call night (15.1% ±7.3%), compared to the pre-call night (28.1% ±12.9%, *P* = .0013) and the on-call duty-free night (26.7% ±11.9%, *P* = .0032). Longer periods of stable sleep occurred during the post-call night (24.2% ±16.3%) and the second-post-call night (24.3% ±15.0%), with no significant differences observed between the post-call nights and the pre-call night or the on-call duty-free night.

Significantly more REM sleep occurred during the on-call night (21.2% ±13.3%), compared with the pre-call night (12.6% ±6.5%, *P* = .0434) and the on-call duty-free night (10.0% ±6.5%, *P* = .0018), and the amount of REM sleep that occurred during the post-call night (19.6% ±26.0%) and second-post-call night (17.5% ±13.0%) were not significantly different from that of the pre-call night and on-call duty-free night. No significant differences in the percentage of unstable sleep were observed among the monitoring periods.

Latency to the first epoch of stable sleep during the on-call night (33.2±44.7 min) and the post-call night (21.1±11.9 min) increased, compared to that of the pre-call night (8.8±6.7 min; *P* = .0313 and.0023, respectively). Latency to the first epoch of stable sleep during the on-call and post-call nights was not significantly greater than that of the on-call duty-free night (13.9±18.5 min; *P* = .090 and.2136, respectively). No significant differences in the onset latency of REM sleep and awakening state or unstable sleep were observed.

## Discussion

The major findings in our current study are the reduced percentage of stable sleep, the increased onset of the latency of stable sleep, and the increased duration of REM sleep during the on-call night shift. This is the first reported application of CPC for the assessment of sleep quality among medical interns and chronic partial-sleep deprivation. Only the total sleep time during the on-call night significantly decreased, compared with that of the on-call duty-free night.

Most studies have shown that stable sleep in the general population is more than 45% [15], [16]. The medical interns in our study had a median percentage of stable sleep of 25.7% during the on-call duty-free night, 26.3% during the pre-call night, and 15.4% during the on-call night. Similar reductions in stable sleep have also been reported in diseases and disorders characterized by impaired autonomic function and poor sleep quality, such as obstructive and central sleep apnea (less than 10%) [23], medication-free major depression (32.5% ±12.0%) [15], fibromyalgia (30.8% ±26.7%) [16], and heart failure with left ventricular ejection fraction (24% ±8%) [17].

The decreased stable sleep and increased REM sleep during on-call night are consistent with previous reports of increased sympathetic activity and reduced vagal tone in acute sleep deprivation [24]. The association between parasympathetic modulation and physiologically stable sleep is represented by high-frequency coupling between heart rate and respiration [13]. Reduced vagal tone and heightened sympathetic activity are known to be associated with an increased risk of CVD [25]. Stable sleep is associated with stable respiration and hemodynamics, and may protect against arrhythmias. Thus, reduced high-frequency coupling during sleep in medical interns may indicate a long-term risk factor for adverse cardiovascular events. In addition to sleep fragmentation, the additional work hours during the on-call night may have also contributed to the reduction in stable sleep during the on-call night. Our previous study showed that an increased score for the Stanford Sleepiness Scale at 12∶00 am on the on-call duty night was a more reliable indicator of sleepiness attributable to a heavy workload, rather than to sleep deprivation, which is more reliably assessed on the post-call morning [12].

Our previous study demonstrated that, during on-call duty sleep periods, increased high-frequency power in HRV occurs from the post-call night to the pre-call night, which contrasted with the decreased ratio of low-frequency to high-frequency power (LF/HF) that persisted throughout the on-call and the on-call duty-free periods [12]. Our inconsistent findings demonstrate the differences between spectrographic coupling metrics and conventional spectral analysis of HRV resulting from the incorporation of both respiration and heart rate signals for measuring the extent of coupling in CPC analysis. The low correlations between CPC-derived sleep indices and spectral HRV measures in patients with major depression also suggest an independent role for CPC analysis in the quantification of sleep physiology [15].

The high-frequency coupling that indicated stable sleep in our current study decreased during the on-call night as a short-term effect of the on-call duty, which differed from the long-term, persistent reduction in the LF/HF of HRV over the entire 3-month internal medicine course. The LF/HF in HRV is related to electroencephalogram (EEG) slow-wave magnitude during sleep [26]. Although the high-frequency coupling in CPC analysis is highly associated with non-cyclic alternating pattern (CAP) sleep, it is not correlated to slow-wave EEG activity [13]. Thus, slow-wave EEG activity and non-CAP sleep are likely independent indicators of deep, high-quality sleep.

In our study, the 10 monthly on-call cycles produced chronic sleep debt in our cohort of interns, and they were unable to recover during the intervals between on-call shifts, thus producing rebounding slow-wave sleep that was accompanied by lower LF/HF values for HRV. The CPC analysis showed that high-frequency coupling was lower than that for the general population, and significant decreases occurred during the on-call night, indicating a role for the autonomic nervous system in responses to chronic and acute sleep deprivation and stressful working conditions.

### Limitations

Our findings have several limitations. The participants were all male, and the sample size was relatively small. Our previous research with medical interns had demonstrated sex differences in cardiac autonomic modulation [27], and cardiac autonomic modulation is known to be affected by the menstruation cycle and other sex differences [28], [29], [30]. Thus, these effects may have been confounding factors in our assessment of the effects of on-call duty. Because we anticipated that increased variability in our assessment of autonomic modulation would result from the inclusion of female interns in a small sample, we did not recruit female participants for our study. In addition, polysomnography, such as EEG, electromyogram, and electrooculogram, were not performed to avoid the interruption of the interns’ clinical practices. Thus, exact correlations with conventional sleep indices were not obtained for our cohort. Furthermore, despite applying an actigraph to identify sleep and awakening status, we were unable to precisely distinguish between REM sleep and the micro-arousal state or periods of quite wakefulness. It’s possible that increased REM during on-call night was due to quite wakefulness in anticipation of the next call. Future study is warranted to validate the nature of very-low-frequency coupling using polysmnography data. In addition, the actual awakenings were also difficult to determine definitely, therefore, the analysis didn’t control for the numbers and duration of awakenings.

### Conclusion

In conclusion, our present findings suggest that medical interns suffer disrupted sleep stability and continuity during on-call night shifts, as quantified by ECG-based CPC analysis. Despite the lack of comparative data from standard polysomnography, our study nonetheless provides a unique viewpoint on sleep stability in the context of cardiovascular physiology. This reliable ECG-based method provides a simple and objective means to evaluate sleep quality in medical staff performing shift work under stressful conditions.
